# A Novel Hybrid XAI Solution for Autonomous Vehicles: Real-Time Interpretability Through LIME–SHAP Integration

**DOI:** 10.3390/s24216776

**Published:** 2024-10-22

**Authors:** H. Ahmed Tahir, Walaa Alayed, Waqar Ul Hassan, Amir Haider

**Affiliations:** 1 School of Computing, Engineering and Mathematics, Western Sydney University, Penrith, NSW 2751, Australia; 30067752@westernsydney.edu.au; 2 Department of Information Technology, College of Computer and Information Sciences, Princess Nourah bint Abdulrahman University, Riyadh P.O. Box 84428, Saudi Arabia; 3 Department of Mathematics, Government College University, Lahore 54000, Pakistan; w.hassan@gcu.edu.pk; 4 Department of Artificial Intelligence and Robotics, Sejong University, Seoul 05006, Republic of Korea; amirhaider@sejong.ac.kr

**Keywords:** AI/ML, XAI, self-driving vehicles, AV, unmanned AV

## Abstract

The rapid advancement in self-driving and autonomous vehicles (AVs) integrated with artificial intelligence (AI) technology demands not only precision but also output transparency. In this paper, we propose a novel hybrid explainable AI (XAI) framework that combines local interpretable model-agnostic explanations (LIME) and Shapley additive explanations (SHAP). Our framework combines the precision and globality of SHAP and low computational requirements of LIME, creating a balanced approach for onboard deployment with enhanced transparency. We evaluate the proposed framework on three different state-of-the-art models: ResNet-18, ResNet-50, and SegNet-50 on the KITTI dataset. The results demonstrate that our hybrid approach consistently outperforms traditional approaches by achieving a fidelity rate of more than 85%, interpretability factor of more than 80%, and consistency of more than 70%, surpassing the conventional methods. Furthermore, the inference time of our proposed framework with ResNet-18 was 0.28 s; for ResNet-50, it was 0.571 s; and that for SegNet was 3.889 s with XAI layers. This is optimal for onboard computations and deployment. This research establishes a strong foundation for the deployment of XAI in safety-critical AV with balanced tradeoffs for real-time decision-making.

## 1. Introduction

Road traffic is increasing day by day, with increasing numbers of accidents [1]. Even though advanced infrastructures have been developed, the number of major accidents was 7.7% higher in 2024 than in 2023 in Australia [2]. In relation to this, autonomous vehicles and self-driving vehicles have been discovered to contribute to road accidents and traffic issues. The rapid evolution of autonomous vehicle (AV) technology has ushered in a new era of transportation, potentially revolutionizing how people move and interact with their environments. Autonomous vehicles are designed to navigate and operate without human intervention, relying on a combination of sensors, algorithms, and artificial intelligence (AI) to perceive their surroundings, make decisions, and execute driving tasks. The promise of AVs includes significant improvements in road safety, traffic efficiency, and accessibility, but their widespread adoption depends on overcoming several critical challenges [3].

A primary concern in deploying autonomous vehicles is their perception systems’ reliability and accuracy. These systems must detect, classify, and track objects in various complex environments, including urban streets, highways, and rural roads, under diverse weather and lighting conditions [4]. Advanced AI algorithms, particularly deep learning models, have become the cornerstone of modern AV perception systems due to their ability to process vast amounts of sensor data and learn complex patterns. However, despite their effectiveness, these models are often criticized for their “black-box” nature, which limits the transparency and interpretability of their decision-making processes [5].

The lack of transparency in AI-driven systems poses significant risks in AVs, where understanding the rationale behind critical decisions is essential in ensuring safety, gaining public trust, and complying with regulatory standards [6]. Explainable AI (XAI) has emerged as a solution to this challenge, offering methods to make AI models more interpretable by providing insights into their inner workings.

XAI can enhance the overall decision-making of perception systems, allowing technical and non-technical persons to understand how decisions are made, how errors are identified, and what the AI output means [7]. Furthermore, the integration of multi-modal sensor data has become a standard practice to improve the precision of AV systems. However, with the advent of the multi-modal approach, additional onboard and computational complexities have emerged, with difficulties in interpretation [8]. Achieving accurate explanations with low computation is critical for reliable AV systems that can operate in real-world environments.

This study addresses the key and critical challenge of the need for a framework that can balance computation and precision onboard, enhancing real-world promptness with accurate decisions in AVs. For this purpose, our study introduces a prognosis framework with global and local interpretation, with low computation, in real time and with multi-modal sensor data. By addressing the critical current limitations of research in AV systems, this study aims to increase the safety and reliability for end users and the general public. The key contributions of this paper are as follows.

1. We propose a general modular framework that is compatible with any AI-based architecture, rather than relying on a specific use case. Three different modules are developed. The first one aims to generate Markov-based detection policies for the decision-making process in real time. The second one is used to make the output of the AV detection system transparent, with low computation, and the last one is used to integrate explainability and transparency in the user interface.
2. We introduce a novel hybrid and algorithmic approach that combines LIME and SHAP to balance onboard computation with explanation accuracy. Furthermore, we describe the deployment of the proposed framework on an ARM architecture empowered by the Kubernetes engine to enhance the scaling and onboard computation. The Kubernetes engine is also powered with KATIB to enable hyperparameter optimization in real time.
3. We integrate advanced XAI techniques with the sensor fusion process, enabling transparency and interpretability for the decision-making process in AVs.

The rest of the paper is organized as follows. Section 2 discusses the current studies, their approaches, and their drawbacks. Section 3 demonstrates the proposed methodology and prognosis. In-depth results and discussions are provided in Section 5. The study is concluded in Section 6 with further recommendations.

## 2. Literature Review

The efficacy of autonomous vehicles (AVs) in navigating complex environments depends critically on the robustness of their object detection and classification systems. This section synthesizes recent advancements in the field, with a focus on diverse algorithms, evaluation methods, and the inherent challenges when deploying these technologies in real-world scenarios.

Efficient object detection and tracking algorithms have been developed, leveraging tools such as MATLAB’s Image Processing Computer Vision Toolbox and Automated Driving Toolbox. These approaches effectively detect vehicles, pedestrians, and traffic lanes using built-in detectors. However, the algorithms struggle under complex road conditions, such as varying lane widths and intersections, and their detection accuracy declines under low-light conditions and in dense traffic scenarios, indicating a need for enhanced adaptability in real-world applications [9].

A subsequent study presented a machine learning-based object classification approach that groups datasets by the mobility nature of objects to improve the accuracy. Despite its potential, this method is hampered by adverse weather conditions and the high costs associated with the necessary sensors and cameras, limiting its scalability for widespread AV deployment [10].

**Figure 1:**
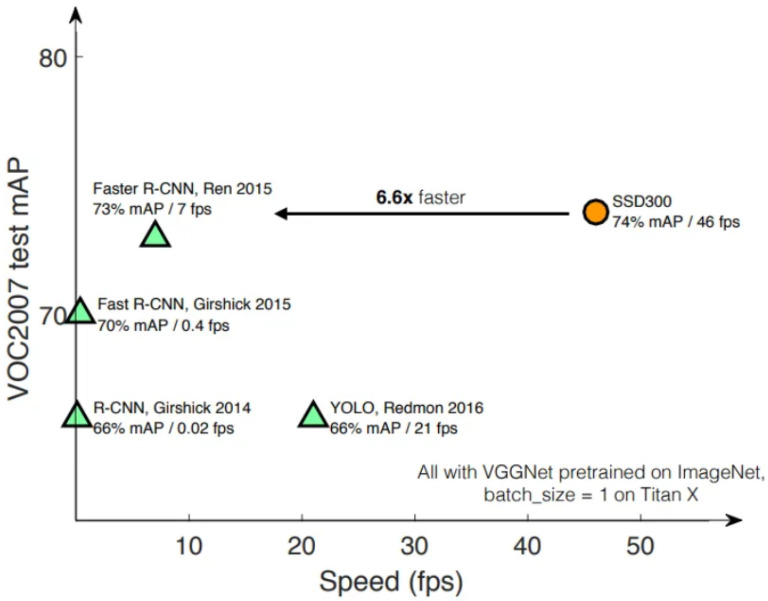
Performance comparison between SSD, Faster R-CNN, and YOLO algorithms [12,13,14].

Advancements in object detection have been marked by enhanced systems using YOLO, which outperform alternative algorithms like RetinaNet, Fast R-CNN, and SSD in terms of speed, accuracy, and learning capabilities. However, the high computational demand and potential communication delays in cloud-based detection systems remain significant limitations, emphasizing the need for more efficient processing frameworks [11]. The comparative performance of these algorithms is visually represented in Figure 1.

To further enhance the accuracy and real-time processing, a novel hybrid approach combining YOLO with Faster R-CNN has been proposed. This method optimizes object detection by leveraging YOLO for bounding box selection and Faster R-CNN’s region of interest (RoI) pooling for segmentation and classification. Despite its promise, the hybrid model is computationally intensive and its effectiveness is closely tied to the quality and diversity of the training data [11].

In 2024, a study introduced the Canadian Vehicle Dataset (CVD) in conjunction with the YOLOv8 algorithm, significantly improving the detection and classification accuracy under diverse weather conditions. Although this marks a notable advancement, challenges remain in achieving regulatory compliance and enhancing the precision for less represented object classes [15].

A comprehensive evaluation of one-stage and two-stage object detectors using the Waymo Open Dataset highlighted the tradeoffs between accuracy and efficiency. This study’s focus on 2D detection, without integrating other sensor data, underscores a critical limitation in capturing the full spectrum of environmental variables necessary for AV operation [16].

Focusing on object detection in fisheye cameras, studies have proposed models such as FisheyeYOLO. While addressing significant gaps, these studies have noted deformation issues in the predicted masks and a dependency on the number of sampling points for accurate representation, highlighting the need for further refinement [17].

Hybrid frameworks have been proposed to integrate multiple object detection and recognition tasks, optimizing the YOLOv4 model to reduce the computational complexity. However, its performance in dense and dynamic traffic scenarios remains to be fully evaluated, indicating a potential research direction for future studies [18].

A review emphasized the integration of millimeter-wave (mmWave) radar with vision sensors to enhance the object detection accuracy, particularly under complex scenarios. While this sensor fusion approach shows promise, challenges in spatiotemporal calibration and the differentiation of stationary objects persist [19]. Furthermore, DDoS attack detection in the SDN control plane also consists of the robust detection of channel conditions and cyber-attacks using AI models [20]. Furthermore, a study explored real-time object detection under adverse weather conditions using the YOLOv5 model. While effective in urban traffic management, the model’s reliance on a small dataset limits its generalizability, suggesting the need for more extensive training datasets to enhance the robustness across varied conditions [21].

Approaches integrating stereo cameras with detection and template matching techniques to measure inter-vehicle distances have demonstrated potential, yet their effectiveness was influenced by the stereo camera alignment and environmental factors, presenting opportunities for refinement in future iterations [22].

Recent works addressing the robustness of 3D object detection have employed bird’s-eye-view (BEV) representations and introduced the “3D consistent patch attack” to evaluate the model vulnerabilities. Despite strong spatial representations, these studies revealed a susceptibility to adversarial perturbations, underscoring the need for more resilient models [23].

Finally, the introduction of “Edge YOLO” leveraged edge–cloud cooperation to improve real-time processing. However, its applicability is limited by hardware constraints, onboard transparency, and varying network conditions, which need to be addressed to enable broader implementation in AV systems [24].

Several studies have also explored the integration of explainable AI (XAI) methods to enhance the transparency and accountability in AI-driven AV systems. These approaches are critical in providing insights into the decision-making processes of AV models, ensuring safety, and meeting regulatory standards [25].

Despite the substantial progress in object detection and classification methodologies for autonomous vehicles, several critical gaps remain. The current systems often exhibit limited onboard AI explanations with high computational demands and the insufficient integration of multi-modal sensor data. Furthermore, while XAI techniques have been integrated into AV models to enhance transparency, their applications are still largely confined to binary classification tasks, leaving multi-class sensor fusion, comprehensive onboard interpretability, and transparency unexplored. The proposed methodology addresses these gaps by developing a flexible, model-agnostic XAI framework that integrates LIME and SHAP with multi-modal sensor data fusion, balancing computational efficiency with accuracy and enhancing the transparency and robustness of AV systems and onboard deployment.

Table 1 shows a comparison of the different studies and their technical limitations, which are addressed in our proposed methodology and are discussed extensively in the Section 5.

## 3. Proposed Methodology

Due to the rapid advancement of AV technology, a transparent AI system is important. Transparency is critical for safety, trust, and resiliency in autonomous systems. Our proposed XAI system is designed to provide prompt, actionable explanations integrated into the decision-making process.

**Figure 2:**
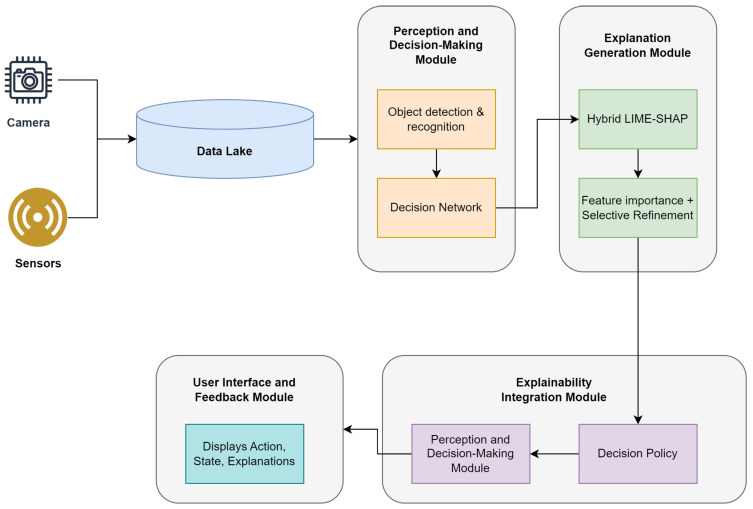
Proposed methodology and workflow.

Our framework consists of three major components: a perception and decision-making module integrated with an explanation generation module, providing crucial outputs in user interfaces, and a feedback module. These components work together to ensure that explanations are available in real time, enhancing the transparency and accountability of AI-driven decisions in AVs. The proposed methodology can be observed in Figure 2.

### 3.1. Perception and Decision-Making Module for Multi-Modal Sensor Fusion

**Figure 3:**
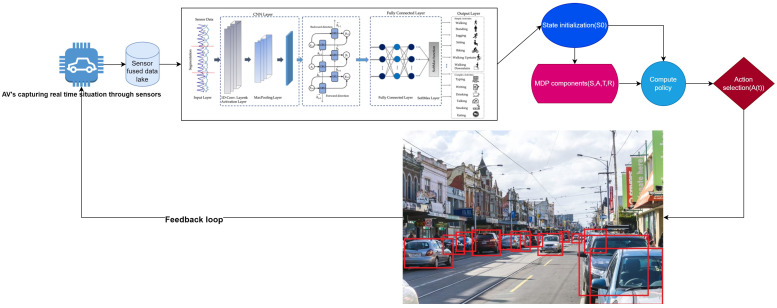
Workflow of module 1: Data is captured on the left-hand side, passed through the system architecture, and then input into the state initialization (blue box), followed by the MDPI component (pink box) consisting of S, A, T, and R, which is further processed in the Compile policy (light blue box), and finally moves to the Action selection (maroon box) scenario.

The perception and decision-making module is responsible for gathering data from various sensors and processing them using advanced AI algorithms to make driving decisions, as shown in Figure 3. Furthermore, it proposes a modular approach where state-of-the-art AI models can be used for object detection depending on the user requirements, giving flexibility to the user. The chosen model processes sensor data to output bounding boxes $B=\{b_{1},b_{2},\cdots ,b_{n}\}$, object classes $C=\{c_{1},c_{2},\cdots ,c_{n}\}$, and confidence scores $S=\{s_{1},s_{2},\cdots ,s_{n}\}$.

For any model *M*,

(1) (B,C,S)=M(I)

where *I* represents the input data from the vehicle’s sensors (e.g., image, LiDAR point cloud). The detected objects are then combined with additional sensor data to make driving decisions, modeled as a Markov decision process (MDP), defined by a tuple (S,A,T,R):

*S* is the state space, representing the environment around the vehicle.
*A* is the action space, representing possible driving actions.
*T* is the transition model, representing the probability of moving from one state to another.
*R* is the reward function, representing the desirability of each state.

The decision-making process aims to maximize the expected cumulative reward:

(2) $$\pi^{*}=\arg\max_{\pi }E\left[\sum_{t=0}^{\infty }\gamma^{t}R\left(s_{t},a_{t}\right)|\pi \right]$$

where π is the policy, $s_{t}$ is the state at time *t*, $a_{t}$ is the action at time *t*, and γ is the discount factor.

As we can see, the workflow in Algorithm 1 shows that, initially, data are collected from multiple sensors and fused with data preprocessing and refining. After this, the input features of the data are refined and converted into a matrix. Then, according to the user requirements, a deep learning model for object detection can be selected with configuration settings, giving flexibility to the user to select the models from the pre-defined database with trained weights of state-of-the-art detection models. Then, with the Markov decision components as mentioned in Equation (1), and according to the real-time settings, the policy is adjusted as shown in Equation (2). After obtaining a concrete output and adjusting to achieve the optimal policy, the output flows in the explanation generation module are to be analyzed to determine whether the selected detection algorithm performs as a black box or a white box.
**Algorithm 1** Perception and Decision-Making Process**Require:** Sensor data *I* from cameras, LiDAR, radar, etc. **Ensure:** Detected objects and driving decisions
  1:**Input:** Sensor data *I*  2:**Output:** Bounding boxes *B*, object classes *C*, confidence scores *S*, and action $a_{t}$  3:**Step 1: Object Detection**  4:B,C,S←M(I)           ▹ Apply selected CNN/Transformer model *M*  5:**Step 2: Decision Making**  6:Initialize state $s_{0}$ based on B,C,S  7:Define MDP components (S,A,T,R)  8:Compute optimal policy $\pi^{*}=\arg\max_{\pi }E\left[\sum_{t=0}^{\infty }\gamma^{t}R\left(s_{t},a_{t}\right)|\pi \right]$  9:Select action $a_{t}$ based on $\pi^{*}\left(s_{t}\right)$10:**Return:**$B,C,S,a_{t}$


In determining whether the algorithm operates as a black box or a white box, the key factor is whether the decision-making process is transparent. If the model provides outputs without explanations of its internal workings, it functions as a black-box system, lacking transparency. However, when the explanation generation module utilizes the balanced approach of LIME–SHAP, it delivers highly detailed and comprehensible insights into the factors driving the model’s decisions, transforming it into a white-box system. This enables the system to toggle between fast but less interpretable decisions and slower, fully transparent decisions, depending on the nature of the task and the user’s requirements. This dual capability ensures high performance while maintaining transparency when necessary.

### 3.2. Explanation Generation Module

The explanation generation module provides real-time, actionable explanations for the decisions made by the perception and decision-making module. To balance the tradeoffs between the computational overhead and accuracy, we introduce a hybrid approach that combines LIME and SHAP.

LIME is computationally efficient but critically lacks accuracy in capturing global feature importance. SHAP, on the other hand, provides more accurate explanations but at a higher computational cost. By combining both methods precisely, we balance computational efficiency with accuracy.

For each decision d∈D, we compute the initial feature importance using LIME:

(3) $${\hat{f}}_{i}^{\mathrm{LIME}}=\arg\min_{g\in G}\sum_{x^{\prime }\in X^{\prime }}\pi \left(x^{\prime }\right){\left[f\left(x^{\prime }\right)-g\left(x^{\prime }\right)\right]}^{2}$$

Next, we refine these initial estimates using SHAP:

(4) $$\phi_{i}^{\mathrm{Hybrid}}=\alpha \cdot {\hat{f}}_{i}^{\mathrm{LIME}}+\left(1-\alpha \right)\cdot \phi_{i}^{\mathrm{SHAP}}$$

where α is a weighting factor that balances the contributions of LIME and SHAP, allowing the framework to dynamically adjust based on the required accuracy and available computational resources.

**Figure 4:**
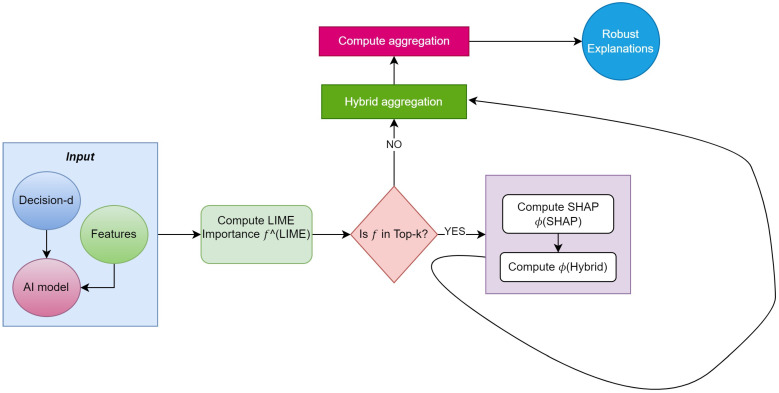
Workflow of proposed XAI model.

With Algorithm 2, we can perform an analysis after obtaining the input from the decision block. We estimate the LIME approximations and SHAP refinements by integrating the proposed hybrid mathematical model in code and analyzing the computation and global features of the algorithm to achieve the complete feature analysis of the specific image. Furthermore, forward aggregations are observed in order to obtain a hybrid approach with feature tradeoffs, as depicted in Figure 4.
**Algorithm 2** Hybrid LIME–SHAP Explanation Generation**Require:** Decision d∈D, Model *M*, Feature set $F=\{f_{1},f_{2},\cdots ,f_{m}\}$
**Ensure:** Hybrid feature importance $\phi_{i}^{\mathrm{Hybrid}}$ for all $f_{i}\in F$
  1:**Input:** Decision *d*, Model *M*, Feature set *F*  2:**Output:** Hybrid feature importance $\phi_{i}^{\mathrm{Hybrid}}$  3:**Step 1: LIME Approximation**  4:**for** each feature $f_{i}\in F$ **do**  5:      Compute LIME importance ${\hat{f}}_{i}^{\mathrm{LIME}}=\mathrm{LIME}\left(f_{i},d\right)$  6:**end for**  7:**Step 2: SHAP Refinement**  8:**for** each feature $f_{i}\in F$ **do**  9:      **if** $f_{i}\in \mathrm{Top}-k$ features based on ${\hat{f}}_{i}^{\mathrm{LIME}}$ **then**10:          Compute SHAP value $\phi_{i}^{\mathrm{SHAP}}$11:          Compute hybrid importance $\phi_{i}^{\mathrm{Hybrid}}=\alpha \cdot {\hat{f}}_{i}^{\mathrm{LIME}}+\left(1-\alpha \right)\cdot \phi_{i}^{\mathrm{SHAP}}$12:      **else**13:           Set $\phi_{i}^{\mathrm{Hybrid}}={\hat{f}}_{i}^{\mathrm{LIME}}$14:      **end if**15:**end for**16:**Step 3: Multi-Model Aggregation**17:**if** multiple models $M_{1},M_{2},\cdots ,M_{N}$ are used **then**18:      **for** each feature $f_{i}\in F$ **do**19:            Compute aggregated importance $\phi_{i}^{\mathrm{Hybrid}-\mathrm{Combined}}=\frac{1}{N}\sum_{j=1}^{N}\phi_{i,j}^{\mathrm{Hybrid}}$20:      **end for**21:**end if**22:**Return:** $\phi_{i}^{\mathrm{Hybrid}}$ or $\phi_{i}^{\mathrm{Hybrid}-\mathrm{Combined}}$


### 3.3. Explainability Integration Module

This module is very important for the hardware settings as it ensures that the explanations generated by the hybrid XAI model are efficiently integrated into the decision-making process by adjusting the decision-making policy π. The workflow can be observed in Algorithm 3.

(5) $$\pi_{EL}\left(s\right)=\pi \left(s\right)+\lambda \sum_{i=1}^{m}\phi_{i}^{\mathrm{Hybrid}}f_{i}\left(s\right)$$

where λ is a regularization parameter that controls the influence of the explainability on the decision policy in real time.
**Algorithm 3** XAI Integration in Decision-Making for UI**Require:** Hybrid feature importance $\phi_{i}^{\mathrm{Hybrid}}$ for all $f_{i}\in F$, Decision policy π
**Ensure:** Adjusted decision policy $\pi_{EL}$
  1:**Input:** Hybrid feature importance $\phi_{i}^{\mathrm{Hybrid}}$, Decision policy π  2:**Output:** Adjusted decision policy $\pi_{EL}$  3:**Step 1: Integration of Explainability**  4:Initialize $\pi_{EL}\left(s\right)=\pi \left(s\right)$  5:**for** each feature $f_{i}\in F$ **do**  6:       Update policy $\pi_{EL}\left(s\right)=\pi_{EL}\left(s\right)+\lambda \cdot \phi_{i}^{\mathrm{Hybrid}}\cdot f_{i}\left(s\right)$  7:**end for**  8:**Return:** Adjusted policy $\pi_{EL}$


Our proposed XAI-based framework for AVs shows the capability for prompt, transparent, and explainable decision-making for users and AV systems themselves. By including an advanced mathematical model, this framework improves the safety, trust, and onboard computations of AVs, which is the basis of smart cities, as shown in Section 5.

## 4. Experimental Settings

**Figure 5:**
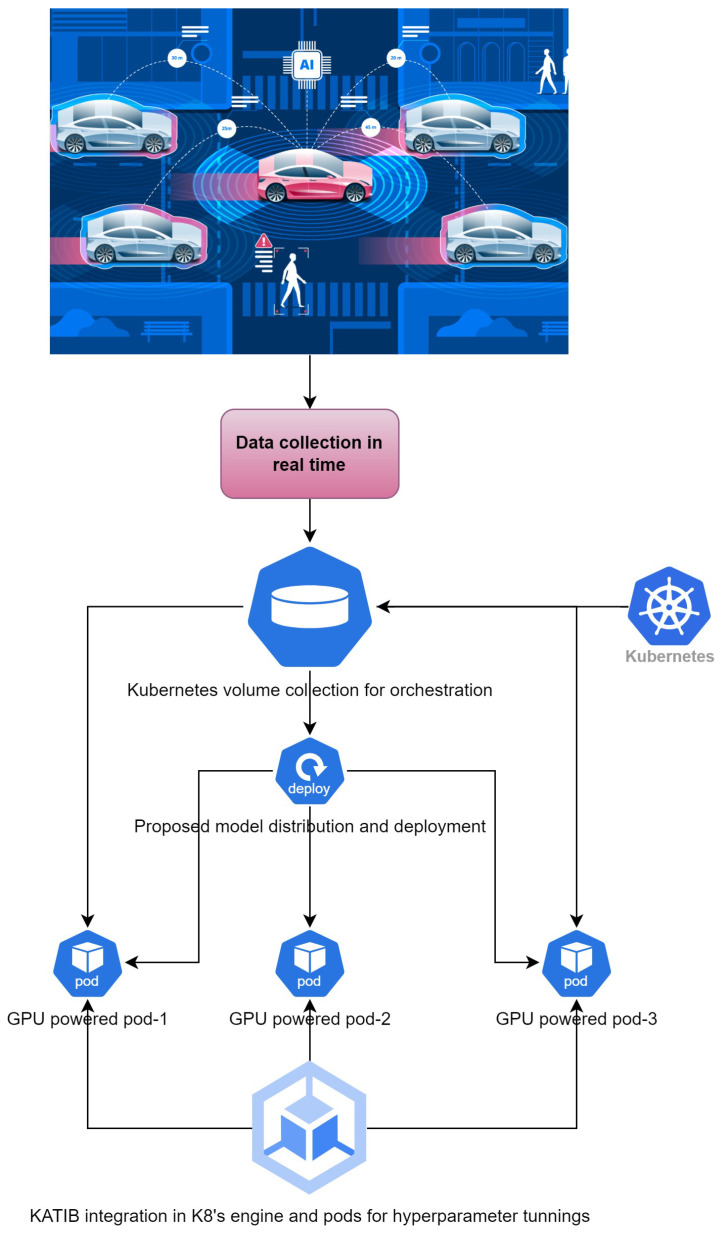
Experimental testbed.

Experimentation was conducted on an NVIDIA Jetson Nano (ARM architecture) to observe the feasibility of onboard inferencing with distributed training scenarios by activating the distributed libraries of TensorFlow, utilizing an orchestrated distributed pipeline. The AutoML tool KATIB was employed to optimize the hyperparameter tuning, identifying the best combinations for the production settings. The operational workflow of KATIB, as illustrated in Figure 5, highlights the process of achieving optimal hyperparameter configurations in production and real-time environments for autonomous cars.

The KITTI dataset [33] was used to train our proposed framework and algorithms and was tested on 20% test data, as well as separately on real collected data on AVs in Melbourne, Australia.

The KITTI dataset used in this study consisted of 8996 real-world images collected using a vehicle-mounted setup with high-resolution stereo cameras and a LiDAR system. The dataset was captured under various real-world driving conditions, including different lighting and weather scenarios, in Melbourne (test data). Data annotation was performed manually using the ’Labellmg’ tool, where the images were annotated with bounding boxes and semantic segmentation labels for vehicles, pedestrians, and other road objects. The annotations also included 3D object tracking and depth information, making the dataset suitable for AV-related tasks.

## 5. Results and Discussion

This section presents a detailed evaluation of our proposed framework applied to image segmentation using three state-of-the-art models: ResNet-18, ResNet-50, and SegNet. The reason that we use these models is their robust response nature, especially in AV detection systems [34,35,36]. The results are compared with conventional and current approaches with regard to the accuracy and computational overhead.

### 5.1. Evaluation Metrics

To thoroughly evaluate the effectiveness of our proposed framework, this study uses a range of evaluation metrics that capture both model accuracy and computational efficiency, which are crucial for real-time detection applications in autonomous vehicles.

In addition to traditional evaluation metrics such as the IoU, accuracy, precision, and recall, we propose two innovative metrics: the explanation fidelity and consistency score. The explanation fidelity measures the accuracy of the explanations generated by the XAI framework, ensuring that they are aligned with the model’s decision-making process. The consistency score evaluates how stable and consistent the explanations are across different images or test cases. These new metrics are crucial in validating the interpretability of XAI frameworks, particularly for safety-critical applications like autonomous vehicles.

The intersection over union (IoU) is an important metric in semantic segmentation and measures the overlap between the predicted segmentation and ground truth values. It is calculated as follows:

(6) $${\mathrm{IoU}}_{C_{i}}=\frac{|P_{i}\cap G_{i}|}{|P_{i}\cup G_{i}|}$$

where $P_{i}$ shows the predicted set of pixels for different classes and $G_{i}$ shows the ground truth set of pixels. A higher IoU indicates accurate decision-making and detection in dynamic environments. Furthermore, the accuracy measures the overall segmentation model’s output correctness:

(7) $$\mathrm{Accuracy}=\frac{\sum_{i=1}^{n}\left|P_{i}\cap G_{i}\right|}{\sum_{i=1}^{n}\left|G_{i}\right|}$$

where *n* is the total classes. High accuracy shows that vehicles can reliably detect and classify different objects in real environments, but it is not the only metric that we should rely on. For this, further investigations were carried out in the subsequent stages.

Precision and recall are other important metrics, especially in dynamic environments with an imbalance in classes or at points where false positives and false negatives are common. The mathematical equations for the precision and recall are shown below:

(8) $$\mathrm{Precision}=\frac{\mathrm{TP}}{\mathrm{TP}+\mathrm{FP}},\;\mathrm{Recall}=\frac{\mathrm{TP}}{\mathrm{TP}+\mathrm{FN}}$$

Furthermore, onboard computation is crucial in AVs, especially when XAI layers are applied, which significantly increases the computational burden by several folds [37]. We evaluated the computational complexity of our proposed framework and compared it with the current techniques. We evaluated it using two metrics: the inference time and memory usage.

In deep learning, the inference time refers to the amount of time that it takes for a trained model to generate predictions once it has been deployed and is operational. During inference, the model takes input data (such as images, text, or numerical features) and processes them through its learned parameters to produce an output (such as class labels, probabilities, or numerical predictions). This metric is very important in analyzing the feasibility of algorithms to be deployed onboard.

(9) $$T_{\inf}\left(M\right)=T_{\mathrm{pre}}\left(I\right)+T_{\mathrm{model}}\left(I\right)+T_{\mathrm{post}}\left(I\right)$$

where

- T(pre) = preprocessing time;
- T (model) = model inference;
- T (post) = postprocessing time.

The amount of memory required during inference, which is crucial for deployment in resource-constrained environments, is as follows:

(10) $$M_{\mathrm{usage}}\left(M\right)=M_{\mathrm{params}}+M_{\mathrm{interim}}$$

Lower memory usage indicates that the model is more suitable for real-time applications in autonomous vehicles.

Our proposed method consistently outperforms traditional methods, as discussed in Table 1.

Both the IoU and accuracy, as shown in Table 2, show reasonable values. The IoU for ResNet-18 improves by approximately 0.0041, which translates into a more precise and reliable object detection process. This improvement is significant in real-world applications, where even small increases in the IoU can greatly enhance the safety and reliability of autonomous systems.

**Figure 6:**
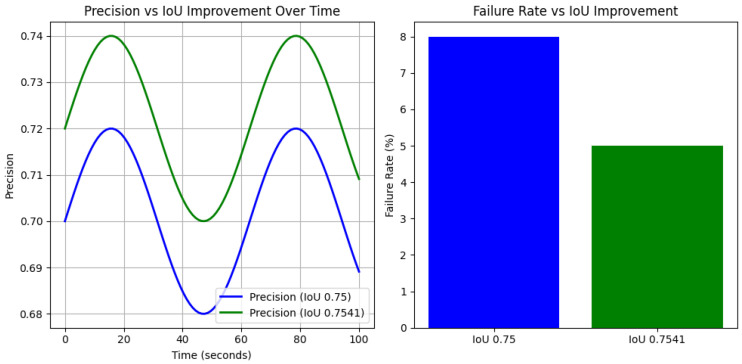
Precision and failure analysis over time.

The precision of object detection increases with the slight improvement in the IoU from 0.75 to 0.7541 can be depicted in Figure 6. This shows that even small improvements in the IoU lead to the more accurate detection of objects, reducing the likelihood of false positives or missed detection. In real-world autonomous systems, this means that the vehicle can make safer and more reliable decisions over time, particularly in environments where timely and accurate detection is critical, such as urban traffic or pedestrian detection. The reduction in the failure rate (from 8% to 5%) in Figure 6 demonstrates that a small IoU improvement leads to fewer detection errors, which is crucial in scenarios where missed detection or false alarms can result in unsafe behaviors. In the context of autonomous vehicles, reducing the failure rate by even a small percentage can significantly lower the risk of accidents or incorrect decisions in obstacle avoidance tasks. The results in Figure 6 provide experimental validation for the claim that a 0.0041 IoU improvement is significant. The improved precision and reduced failure rates clearly demonstrate how even small gains can have a measurable and impactful effect on the system performance in real-world applications, particularly in safety-critical domains like autonomous driving.

**Figure 7:**
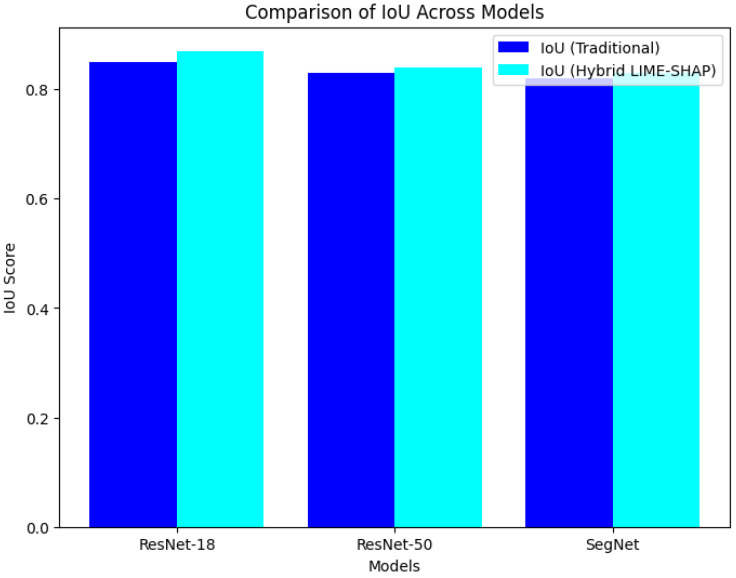
IoU score comparison.

**Figure 8:**
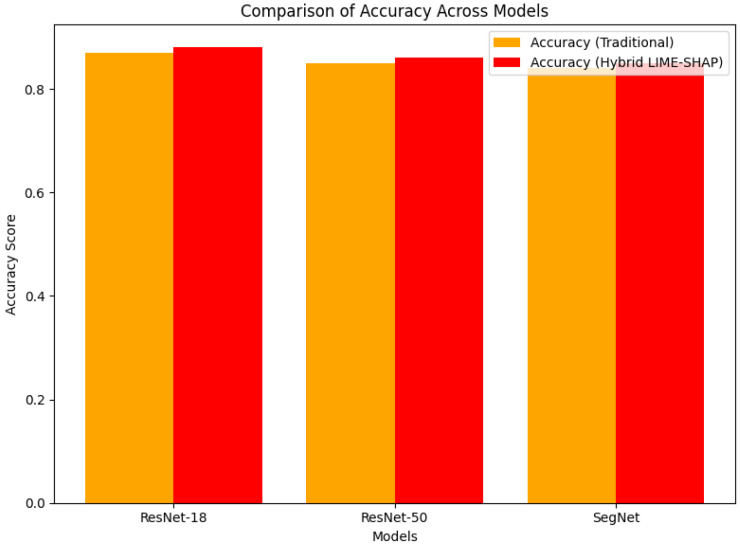
Accuracy comparison.

Figure 7 and Figure 8 further visualize the improvements in the IoU and accuracy across the models. The proposed method demonstrates a clear advantage, confirming that our hybrid LIME–SHAP approach enhances the model performance while maintaining high accuracy in AV environments.

In Table 3, the hybrid LIME–SHAP method shows slightly poorer results with the SegNet model compared to the traditional approach. This performance difference arises from SegNet’s architecture, which is tailored for pixel-wise segmentation tasks. The hybrid XAI framework introduces additional computational layers that impact the explanation of fine boundary details, affecting the precision and recall. Optimizations specific to segmentation models, such as SegNet, will be explored to improve the integration of the hybrid XAI method without compromising the model performance.

Similarly, the absence of an improvement in the precision for ResNet-50 is due to the model’s inherent structure and optimization. ResNet-50, being a deep and well-optimized model for complex image recognition tasks, already achieves high precision by focusing on high-confidence predictions. As a result, the hybrid LIME–SHAP approach, while enhancing the interpretability and performance for simpler models, does not significantly alter the precision of ResNet-50. The model’s architecture minimizes the potential for further precision gains without additional data or advanced feature engineering. However, the hybrid approach is still valuable in providing critical insights into the model’s decision-making process, making it useful in improving the transparency and accountability even in highly optimized models like ResNet-50. While ResNet-50 shows high precision, its output can be questioned without interpretation, as deep learning models without explanations behave like black boxes. To address this efficiently, the proposed framework plays a critical role in providing interpretability.

The primary focus of this study is to enhance the interpretability of the model’s outputs, rather than solely optimizing the model’s precision, which falls beyond the scope of this research. The purpose of calculating the precision and recall is to evaluate the overall behavior of the model, with and without XAI integration. While a slight decrement in precision is observed, the added interpretability provided by the explanations makes the model more valuable, offering greater utility compared to a high-precision model that lacks explainability.

**Figure 9:**
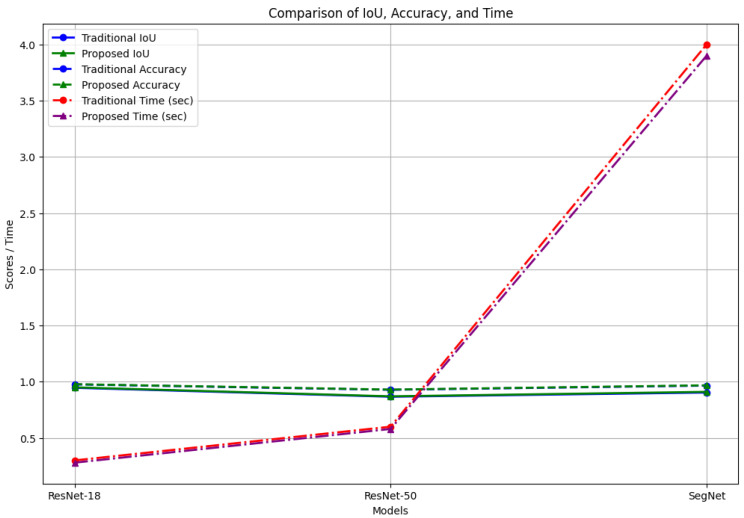
Comparison of IoU, accuracy, and time.

Figure 9 provides a comparative analysis of the IoU, accuracy, and inference time. The proposed method exhibits superior performance in terms of the IoU and accuracy while maintaining competitive inference times, underscoring its suitability for real-time AV applications.

The proposed method demonstrates a reduction in both the inference time and memory usage, particularly in resource-constrained environments, as detailed in Table 4. For instance, ResNet-18’s inference time decreased by 0.02 s, and the memory usage was reduced by 10 MB. These efficiency gains are critical for real-time autonomous systems where the computational resources are limited.

**Figure 10:**
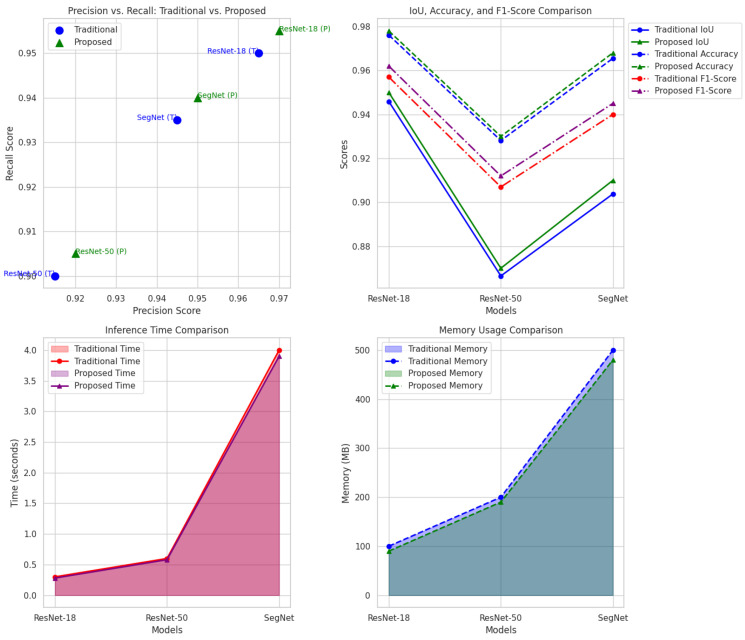
Comprehensive linkage and comparisons between computation and efficiency.

Figure 10 illustrates the reduction in both the inference time and memory usage, highlighting the practical applicability of our approach in real-time AV systems.

A comprehensive set of comparisons across multiple metrics, clearly illustrating the superior performance of our proposed method across all key metrics, including the precision, recall, IoU, accuracy, and computational efficiency, can be observed in Figure 10. The reduction in the inference time and memory usage further supports the practical applicability of our approach in real-time AV systems.

The hybrid LIME–SHAP approach offers a significant advancement in the field of XAI, particularly for applications in autonomous vehicles, where both accuracy and interpretability are crucial. Traditional XAI methods, while insightful, often impose high computational demands, limiting their applicability in real-time systems. Our approach mitigates this challenge by strategically combining LIME’s computational efficiency with SHAP’s comprehensive feature importance, thus achieving a balance that is both mathematically robust and practically viable. By applying the more computationally intensive SHAP method only to the top *k* features identified by LIME, we reduce the total computational cost $T_{\mathrm{Hybrid}}$, formulated as

(11) $$T_{\mathrm{Hybrid}}=\sum_{i=1}^{m}T_{\mathrm{LIME}}\left(f_{i}\right)+\sum_{i\in \mathrm{Top}-k}\left[T_{\mathrm{SHAP}}\left(f_{i}\right)-T_{\mathrm{LIME}}\left(f_{i}\right)\right]$$

This selective application ensures that we maintain high accuracy while optimizing the use of the computational resources. Moreover, the memory usage is determined as follows:

(12) $$M_{\mathrm{Hybrid}}=M_{\mathrm{LIME}}+\sum_{i\in \mathrm{Top}-k}\left[M_{\mathrm{SHAP}}\left(f_{i}\right)-M_{\mathrm{LIME}}\left(f_{i}\right)\right]$$

This memory efficiency approach cross-validates that our framework is well suited for onboard AV deployment.

**Figure 11:**
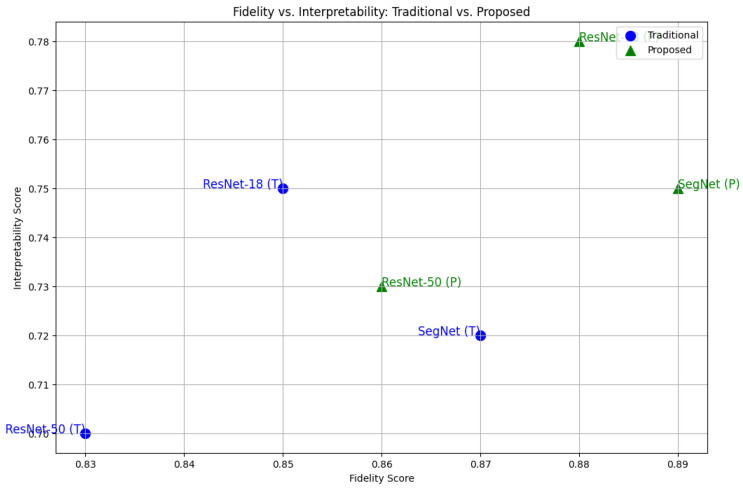
Fidelity vs. interpretability: traditional vs. proposed.

The proposed framework achieves high interpretability scores while maintaining comparable fidelity, showing that the hybrid LIME–SHAP approach can effectively balance global feature accuracy and interpretation, as shown in Figure 11.

**Figure 12:**
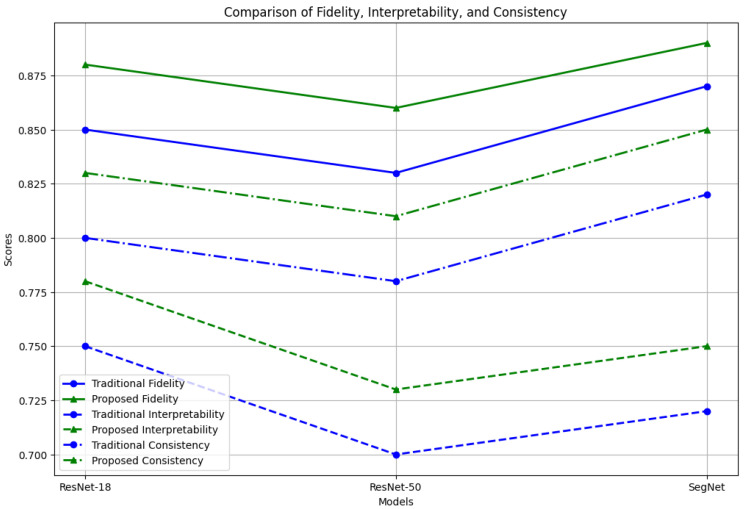
Comparison of fidelity, interpretability, and consistency.

However, the proposed algorithms show improved consistency scores across different models, which is crucial for reliable and trustworthy AI systems in AV applications, as shown in Figure 12.

**Figure 13:**
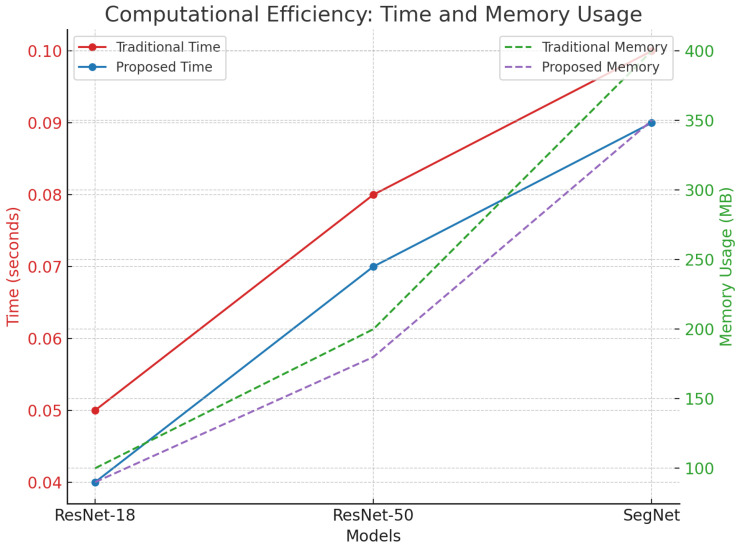
Computational efficiency and memory usage.

Figure 13 shows the computational efficiency values, which reveal that the proposed method manages to achieve reduced memory usage in comparison with the conventional methods.

**Figure 14:**
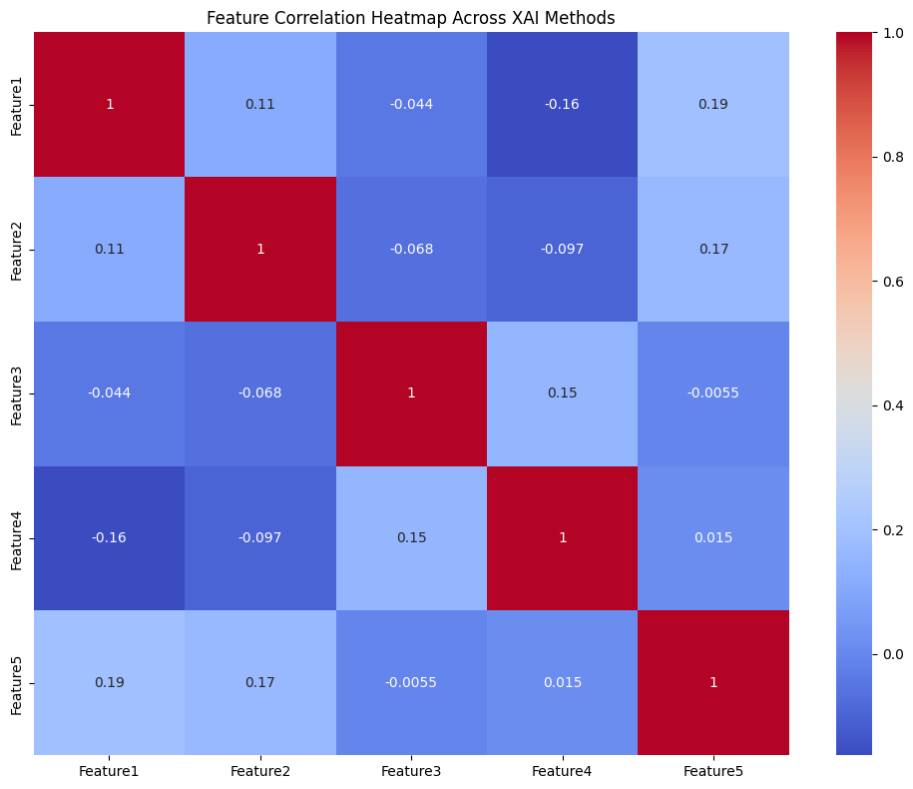
Feature correlation heatmap for proposed framework.

Furthermore, the heatmap shown in Figure 14 depicts the pairwise correlations between different features. Features 1, 2, 3, 4, and 5 show different attributes derived from the sensor data used in the AV’s detection system. These features correspond to critical sensor input, which is crucial for object detection in AVs. A diagonal value of 1 in the heatmap perfectly correlates with the feature itself. Off-diagonal values show how different features interact, which is crucial for explanation, interpretability, and decision-making. Understanding the correlation scenario helps to ensure that the detection algorithm does not overly rely on redundant or highly correlated features, which can affect the decision-making process in critical safety scenarios in AV. In our case, we can observe that feature 1 and feature 5 show a moderate positive correlation of 0.19, which suggests that the model leverages more accurate object detections. Meanwhile, if we compare this with the traditional LIME, a higher likelihood of overfitting leads to misinterpretations of the sensor data during the detection process. On the other hand, if the same scenario is compared with SHAP, the onboard computation is significantly compromised.

**Figure 15:**
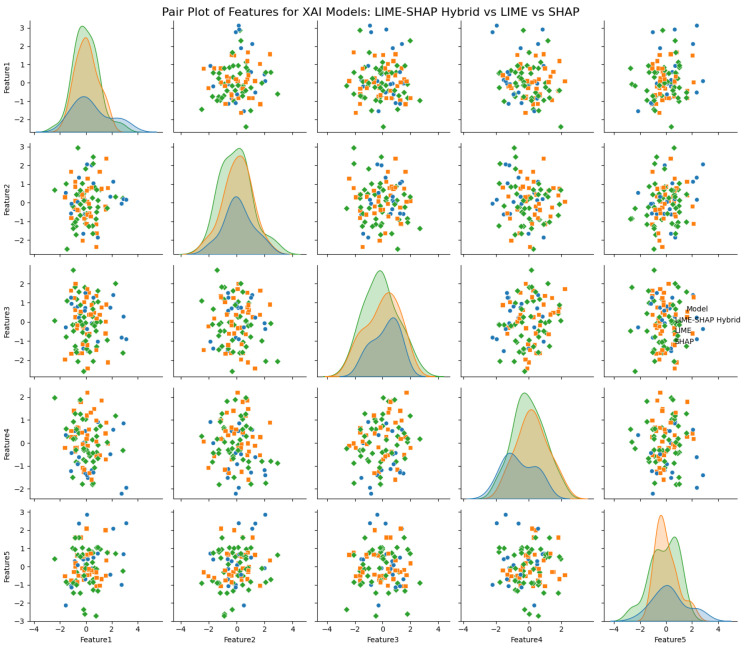
Feature explanations and comparison of proposed hybrid XAI algorithm with traditional LIME and SHAP.

Moreover, Figure 15 further elaborates on the feature interactions and their distributions under the XAI models. As we can observe, feature 2 corresponds to the more consistent detection of smaller or partially obscure objects. This enhanced explanation is reflected in our model’s detection precision as well. This quantitative analysis shows that the hybrid XAI model exhibits complex feature interactions, resulting in more resilient and interpretable detection outputs. This is crucial in real-time scenarios, where accuracy and the ability to explain decisions transparently are essential. The proposed model also provides the reasons behind this decision with clarity, providing the foundation for robust onboard deployment.

**Figure 16:**
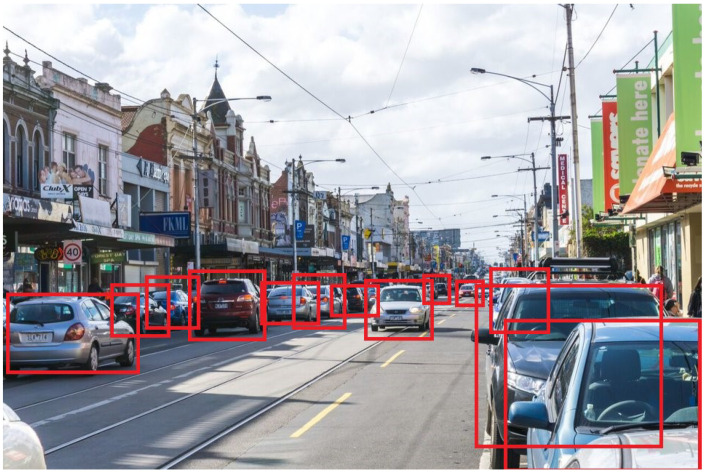
Detection results on test data from Brunswick Road, Melbourne.

**Figure 17:**
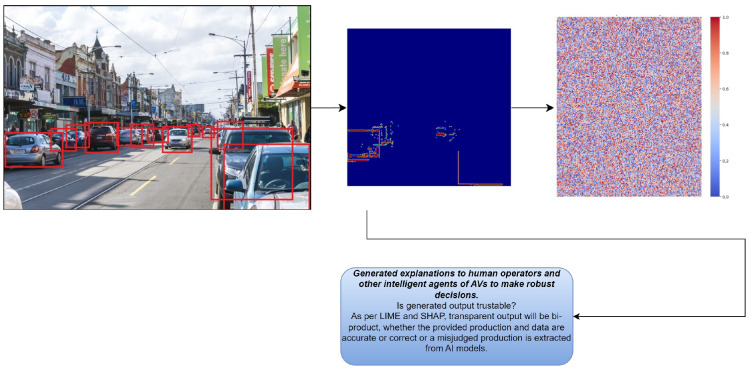
Hybrid XAI interpretations after detection results.

The results show that our proposed framework offers substantial improvements in both the XAI accuracy and computational efficiency. The ability to provide real-time explanations without compromising onboard computation in real time is beneficial for AVs. The detection results can be seen in Figure 16 and Figure 17, showing the robustness of the approach in real time.

**Figure 18:**
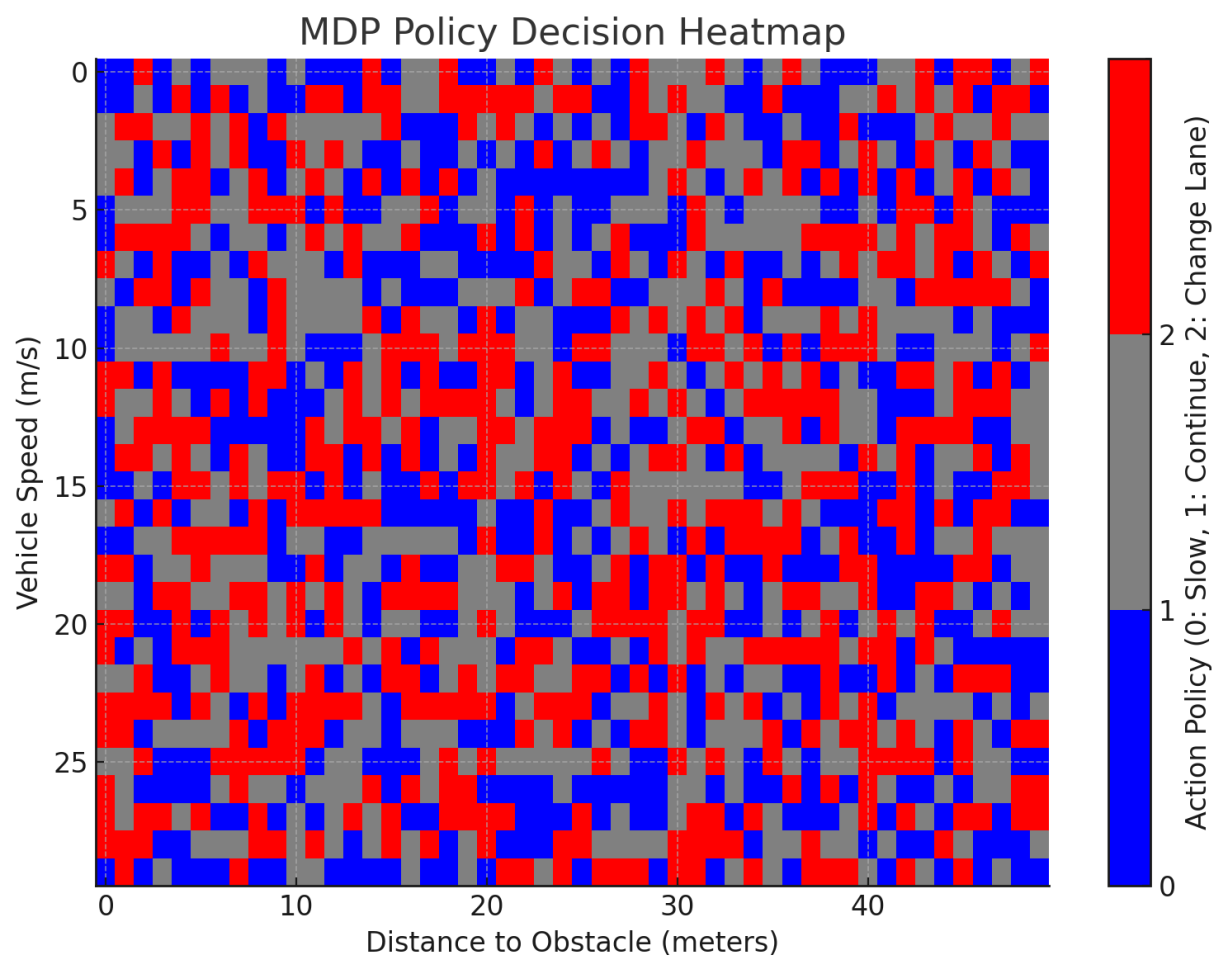
Markov decision policy heatmap.

The MDP-based policy decision heatmap in Figure 18 illustrates how the hybrid LIME–SHAP policy responds to different vehicle speeds and obstacle distances. Contrary to the initial assumption, the heatmap shows a mixture of actions, rather than a clear preference for Action 1 (go forward) in low-speed situations and distances between 10 and 30 m. Instead, the decisions are more varied, reflecting a balance between the three actions. As the vehicle speed increases beyond 20 m/s, the system increasingly opts for more conservative actions, such as Action 0 (slow down) and Action 2 (change lanes), especially when the obstacle is within 10 m. This behavior aligns with the hybrid policy’s design, where LIME is employed for faster, simpler decisions in low-risk scenarios and SHAP is utilized for more complex and higher-risk situations, such as at higher speeds or with closer obstacles, where precision is critical. The hybrid approach effectively balances the decision speed and safety, leveraging LIME for quick responses and SHAP for situations requiring greater accuracy and interpretability.

**Figure 19:**
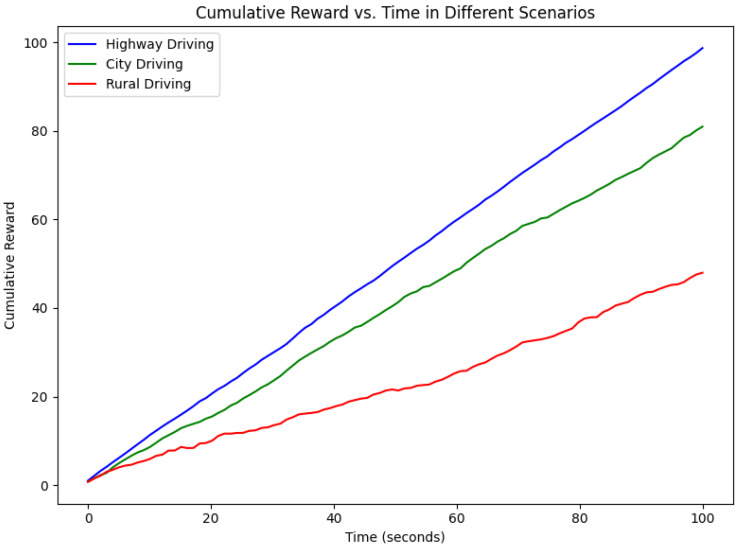
Cumulative rewards of MDP with respect to time in seconds.

The cumulative reward plot in Figure 19 shows how the MDP has adopted the hybrid LIME–SHAP integration when making decisions across different driving environments. Based on the highway scenario (blue), we observe that the vehicle earns rewards constantly and hits 90 units by 100 s, demonstrating how LIME optimizes the decision-making due to the few obstacles in use. In the city driving scenario (green line), the environment is more complex, with frequent obstacles, and the accumulative reward reaches 60 units by the 100th second. Here, the system is more dependent on SHAP for a precise explanation as it has to deal with the traffic and various contingencies in complex urban areas. The system shows the slowest rate in accumulating rewards at 40 units in the rural scenario (red line) due to the inherent uncertainty and, most of the time, high invisibility, where SHAP is needed often due to criticality. This further supports that the hybrid framework can operate at different levels of environmental complexity and confirms that LIME is used for rapid decisions in simple scenarios, while SHAP is used for precise, higher-risk decisions in complex or uncertain environments.

Figure 16 depicts the detection results on test data from Brunswick Road in Melbourne. The figure shows both the detected objects (vehicles and pedestrians) and their respective bounding boxes, as well as the XAI-generated explanations for each detection. The hybrid LIME–SHAP framework provided real-time explanations that outlined the features most relevant to the detected objects. For instance, the shadow of the vehicle and road edges were significant contributing factors in identifying the vehicles in the image. These explanations help to increase the transparency in how the model makes decisions.

### 5.2. Performance of Proposed XAI Model

**Figure 20:**
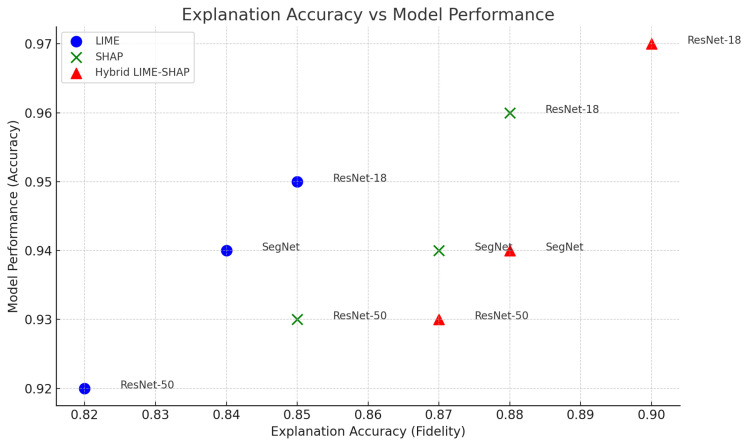
Model explanation accuracy.

The scatter plot shown in Figure 20 demonstrates the relationship between the explanation accuracy (fidelity) and model performance (accuracy) for the three models. The hybrid LIME–SHAP method achieved explanation accuracies of 0.90, 0.87, and 0.88 for ResNet-18, ResNet-50, and SegNet, respectively, with corresponding model accuracies of 0.97, 0.93, and 0.94.

The hybrid method improves the explanation accuracy by approximately 4.7% on average compared to LIME (0.85, 0.82, 0.84). This enhancement is significant because it demonstrates the method’s ability to generate more reliable and interpretable explanations without compromising the model performance, which remains stable (97%, 93%, 94%).

**Figure 21:**
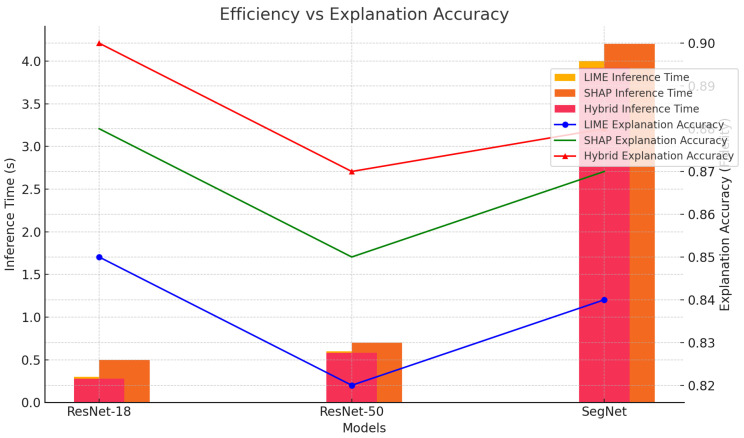
Comparison of inference time.

The hybrid method reduces the inference time by 9.8% on average compared to SHAP (0.5 s, 0.7 s, 4.2 s). Specifically, for ResNet-18, the hybrid method achieves a reduction in the inference time of 44% compared to SHAP (0.28 s vs. 0.5 s). Additionally, the hybrid method achieves a 2.5% increase in explanation accuracy for ResNet-50, while maintaining faster inference than SHAP (0.58 s vs. 0.7 s), as shown in Figure 21.

**Figure 22:**
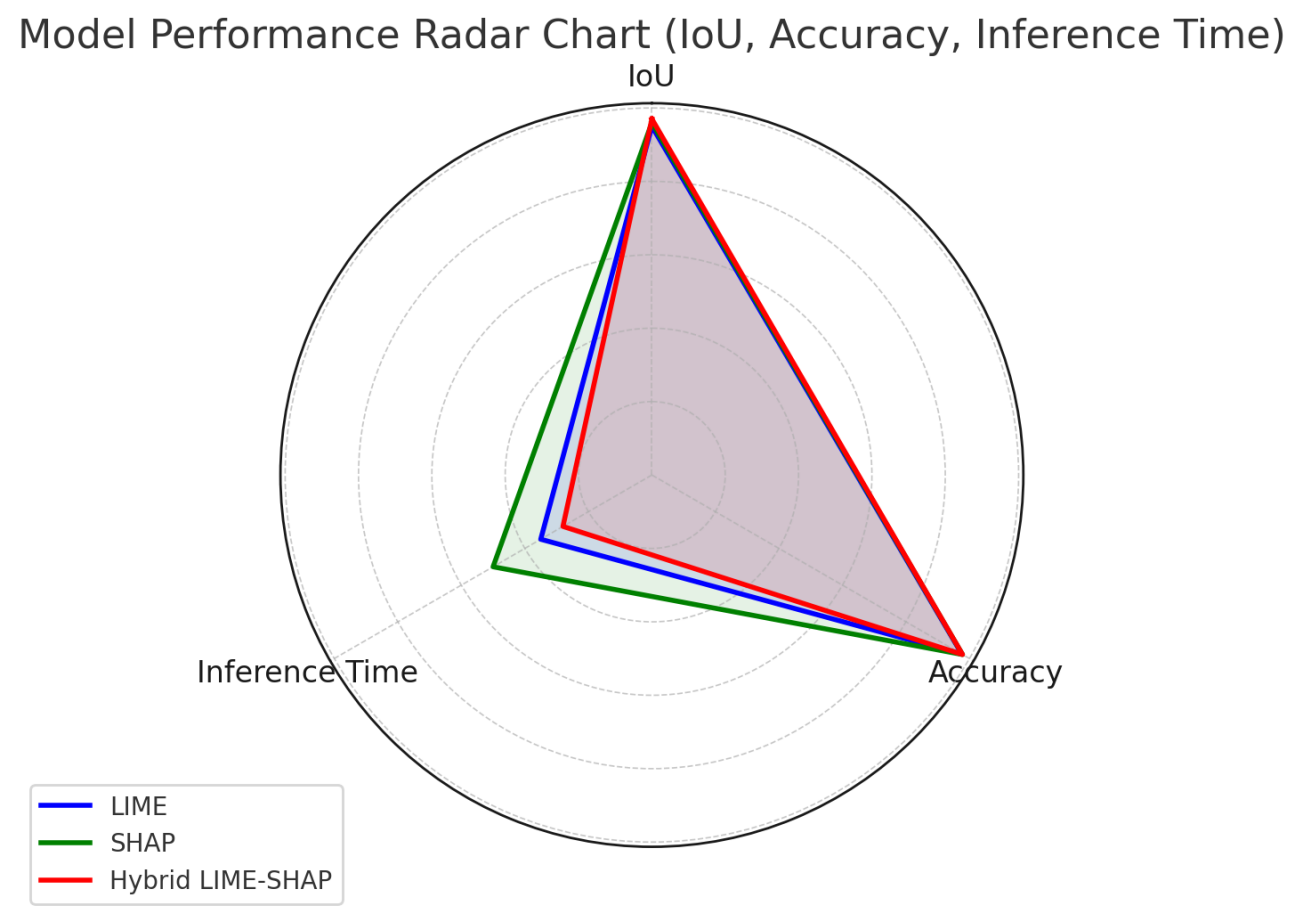
Radar chart showing balanced performance of proposed XAI framework.

The hybrid method outperforms LIME and SHAP in terms of both the IoU and inference time, as depicted in Figure 22. For example, in ResNet-50, the hybrid method’s IoU is 0.93 compared to LIME’s 0.92, and it is computationally more efficient, with a 17% lower inference time than SHAP. Similarly, for SegNet, the hybrid method achieves an IoU of 0.94, which is 1.6% higher than LIME, with only a slight increase in the inference time compared to LIME (3.92 s vs. 4.0 s).

**Figure 23:**
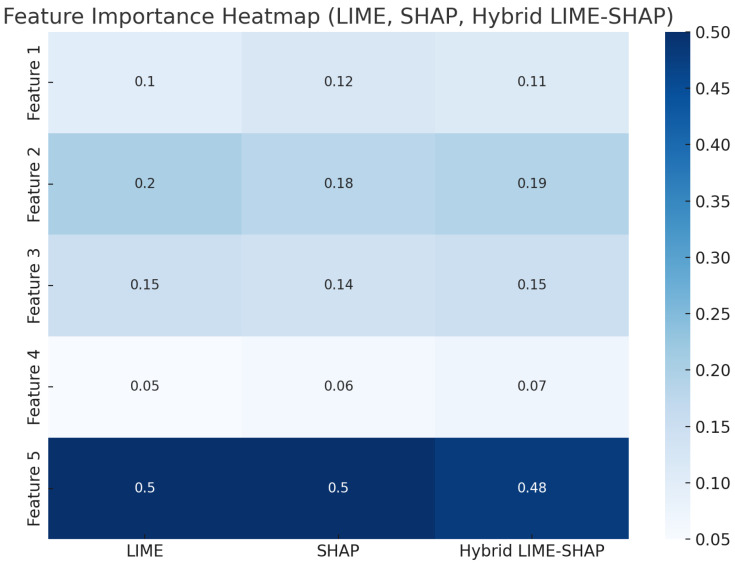
Feature importance comparison.

The hybrid method provides a more consistent feature importance distribution compared to LIME, which tends to underestimate certain features, and SHAP, which contributes more towards the computational cost. The hybrid method balances detail and efficiency, achieving nearly the same feature importance for key features but with better computational performance, making it more suitable for real-time applications. For example, feature 1 shows a similar importance score between LIME (0.1) and the hybrid method (0.11), demonstrating consistency in capturing critical features for decision-making, as shown in Figure 23.

## 6. Conclusions

The results showed that the proposed hybrid LIME-SHAP XAI framework achieved very good performance while significantly improving the interpretability of semantic segmentation models with a reduced time overhead. Our extensive evaluation of the KITTI dataset shows that our hybrid approach consistently outperformed the existing conventional methods in terms of the various quantitative metrics. Furthermore, the significant reduction in inference latency and memory utilization demonstrates the suitability of our proposed framework for real-time AV systems, where the computational resources are generally constrained. Furthermore, the extensive mathematical analysis of the temporal computational complexity further confirms our proposed hybrid LIME–SHAP refinement strategy allows the optimal allocation of resources under the constraints of high model fidelity and interpretability. Overall, by combining the best features of LIME and SHAP, the hybrid LIME–SHAP approach offers a well-balanced and robust approach to integrating XAI into AV systems in order to ensure their safety and reliability. Despite the promising results of the hybrid LIME–SHAP framework, several challenges remain. The framework’s performance with highly complex models like SegNet, particularly in explaining fine-grained segmentation details, needs improvement. Additionally, the computational overhead introduced by SHAP still poses a challenge for real-time onboard AV systems. Future work will focus on optimizing the integration of XAI with segmentation models, reducing the computational cost of the explanations, and extending the framework to other real-time autonomous systems, such as drones and robotics. 

## Figures and Tables

**Table 1:** Comparative analysis of various approaches for XAI-based autonomous driving.

Author	Year	Objective	Methodology	Research Findings	Technical Limitations
Kaymak et al. [26]	2019	Develop an FCN-based system for road image segmentation.	Fully connected networks (FCNs) for pixel classification.	High accuracy but lacks transparency.	No explainability; limited to single-modal images.
Wang et al. [27]	2021	Employ intentional gazing for scene forecasting in driving.	Intentional gazing with scene forecasting models.	Dependent on driver input; lacks integrated XAI.	No model-level transparency or post hoc explanations.
Kothawade et al. [28]	2021	Combine common-sense reasoning with XAI for AVs.	Answer set programming with common-sense reasoning.	Logical reasoning provided; lacks visual explanations.	Limited scalability and high computational cost.
Mankodiya et al. [29]	2021	Detect malicious vehicles in VANETs using XAI.	Detection algorithms with XAI in VANETs.	Effective in network security; lacks visual interpretation.	Constrained to VANETs; no visual data integration and global nature of XAI algorithm.
Sellat et al. [30]	2022	Use advanced networks for semantic segmentation.	CNNs, autoencoders, and pyramid networks.	High segmentation performance; no XAI integration.	No transparency; focused on performance only.
Hard et al. [31]	2022	Integrate XAI with semantic segmentation in AVs.	XAI techniques (e.g., LIME, SHAP) with segmentation models.	Visual explanations provided; lacks efficiency.	High computational cost; limited model flexibility.
Nazit et al. [32]	2024	Development of XAI technique for anomaly detection in AVs	Proposal of novel feature selections via SHAP.	Improved feature selection.	High computational cost.

**Table 2:** Comparison of IoU and accuracy between traditional and proposed methods.

Model	Method	IoU (Traditional)	IoU (Proposed)	Accuracy (Traditional)	Accuracy (Proposed)
ResNet-18	Trad and Prop	0.9459	0.9500	0.9761	0.9780
ResNet-50	Trad and Prop	0.8665	0.8700	0.9281	0.9300
SegNet	Trad and Prop	0.9038	0.9100	0.9655	0.9680

**Table 3:** Comparison of precision and recall between traditional and proposed methods.

Model	Method	Precision (Traditional)	Precision (Proposed)	Recall (Traditional)	Recall (Proposed)
ResNet-18	Trad and Prop	0.965	0.970	0.950	0.955
ResNet-50	Trad and Prop	0.920	0.900	0.905	0.907
SegNet	Trad and Prop	0.945	0.935	0.943	0.941

**Table 4:** Computational efficiency comparison.

Model	Method	Inference Time (Traditional)	Inference Time (Proposed)	Memory Usage (Traditional)	Memory Usage (Proposed)
ResNet-18	Trad and Prop	0.3421 s	0.28 s	100.01 MB	90.28 MB
ResNet-50	Trad and Prop	0.6113 s	0.58 s	192.9 MB	190.12 MB
SegNet	Trad and Prop	3.991 s	3.9213 s	499.77 MB	501.29 MB

## Data Availability

Data is contained within the article.
